# Icariside II Attenuates Methamphetamine-Induced Neurotoxicity and Behavioral Impairments via Activating the Keap1-Nrf2 Pathway

**DOI:** 10.1155/2022/8400876

**Published:** 2022-03-28

**Authors:** Jian Huang, Jiuyang Ding, Zhuo Wang, Yanning Li, Yitong He, Xiaohan Wang, Haoliang Fan, Qiqian Xie, Pingming Qiu

**Affiliations:** ^1^School of Forensic Medicine, Southern Medical University, Guangzhou 510515, China; ^2^School of Forensic Medicine, Guizhou Medical University, Guiyang 550004, China; ^3^Department of Infertility and Sexual Medicine, The Third Affiliated Hospital of Sun Yat-sen University, Guangzhou 510630, China

## Abstract

Chronic and long-term methamphetamine (METH) abuse is bound to cause damages to multiple organs and systems, especially the central nervous system (CNS). Icariside II (ICS), a type of flavonoid and one of the main active ingredients of the traditional Chinese medicine *Epimedium*, exhibits a variety of biological and pharmacological properties such as anti-inflammatory, antioxidant, and anticancer activities. However, whether ICS could protect against METH-induced neurotoxicity remains unknown. Based on a chronic METH abuse mouse model, we detected the neurotoxicity after METH exposure and determined the intervention effect of ICS and the potential mechanism of action. Here, we found that METH could trigger neurotoxicity, which was characterized by loss of dopaminergic neurons, depletion of dopamine (DA), activation of glial cells, upregulation of *α*-synuclein (*α*-syn), abnormal dendritic spine plasticity, and dysfunction of motor coordination and balance. ICS treatment, however, alleviated the above-mentioned neurotoxicity elicited by METH. Our data also indicated that when ICS combated METH-induced neurotoxicity, it was accompanied by partial correction of the abnormal Kelch 2 like ECH2 associated protein 1 (Keap1)-nuclear factor erythroid-2-related factor 2 (Nrf2) pathway and oxidative stress response. In the presence of ML385, an inhibitor of Nrf2, ICS failed to activate the Nrf2-related protein expression and reduce the oxidative stress response. More importantly, ICS could not attenuate METH-induced dopaminergic neurotoxicity and behavioral damage when the Nrf2 was inhibited, suggesting that the neuroprotective effect of ICS on METH-induced neurotoxicity was dependent on activating the Keap1-Nrf2 pathway. Although further research is needed to dig deeper into the actual molecular targets of ICS, it is undeniable that the current results imply the potential value of ICS to reduce the neurotoxicity of METH abusers.

## 1. Introduction

Methamphetamine (METH), an amphetamine-type stimulant that appears as white transparent irregular crystals, commonly known as “ice,” is rapidly and widely abused across the world due to easy access to raw materials, simple synthetic methods, and low production costs [1]. The *World Drug Report 2020* issued by the United Nations Office on Drugs and Crime pointed out that in the past year, about 27 million people worldwide have used amphetamine-type stimulants including METH. METH is known for its strong excitability and high potency for addiction, which can lead to repeated withdrawal and relapse. Some researchers defined it as a chronic recurrent disease characterized by compulsive drug taking, inability to control intake, and strong drug cravings [2]. Chronic and long-term METH abuse is bound to cause serious toxic effects on abusers ending with multiple organ and system damages. Especially, the pathological changes of the central nervous system (CNS) result in mental disorders such as psychosis, anxiety, and depression [3, 4] and in cognitive dysfunctions such as impairment of executive function, attention disability, and working memory loss [2, 5]. Nevertheless, there is still a lack of reliable and effective drugs to prevent and treat METH-induced neurotoxicity.

Dopaminergic neurons are one of the main attacked targets by METH, and its damage caused by METH is similar to the pathological changes of Parkinson's disease (PD). While the mechanism of METH-induced neurotoxicity is not well understood, our previous studies and those of other researchers' works have indicated that involves the regulation of neuronal excitotoxicity, oxidative stress, neuroinflammation, apoptosis, and autophagy [6–11]. Among these molecular and cellular processes, oxidative stress seems to be a bridge connecting neuroinflammation, apoptosis, and autophagy [4, 12, 13]. When oxidative stress occurs, the endogenous antioxidant system is also activated, thereby inhibiting the injury from the oxidative stress and keeping the body in a balanced state. The pathway involving Kelch 2 like ECH2 associated protein 1 (Keap1) and nuclear factor erythroid-2-related factor 2 (Nrf2) is crucial in the endogenous antioxidant system. Nrf2 is a redox-sensitive transcription factor containing the basic structure of the leucine zipper [14]. Under physiological conditions, Nrf2 mainly concentrates in the cytoplasm and forms a complex with Keap1, which could mediate the degradation of Nrf2 through the ubiquitin-proteasome system [14, 15]. When reactive oxygen species (ROS) or other substances stimulate, Nrf2 will dissociate from Keap1 and enter the nucleus, where it interacts with the antioxidant-response element and activates the expression of antioxidant enzymes such as heme oxygenase 1 (HO-1) and NAD(P)H:quinone oxidoreductase 1 (NQO-1) [14, 15]. However, when the damage is severe enough to exceed the regulatory range, the Keap1-Nrf2 pathway will also be affected and lead to a vicious circle of oxidative stress. Recent studies have confirmed that the Keap1-Nrf2 pathway dysfunction plays an important role in METH-induced neurotoxicity [16–19].

Icariside II (ICS), a type of flavonoid, is one of the main active ingredients of the traditional Chinese medicine *Epimedium* [20]. Extensive data have shown that ICS could exhibit a variety of biological and pharmacological properties, including anti-inflammatory [21, 22], antioxidant [23, 24], and anticancer [20, 25] activities. Recent studies have also found that ICS has a protective effect against CNS damage. For example, ICS could attenuate lipopolysaccharide-induced amyloid production and inflammation in astrocytes [21] and suppress oxygen-glucose deprivation/reperfusion- (OGD/R-) induced hippocampal neuronal death [26]. However, there are no research reports on whether ICS could protect against METH-induced neurotoxicity. And if it does so, the relevant molecular mechanisms are worthy of further study.

To verify this hypothesis, we observed the intervention effect of ICS and investigated the potential influence of the Keap1-Nrf2 pathway in a chronic METH-abuse mouse model that developed CNS injury.

## 2. Materials and Methods

### 2.1. Reagents

**Figure 1:**
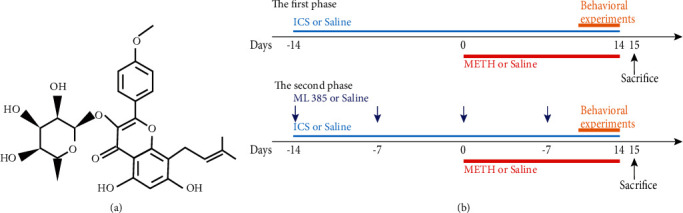
(a) Molecular structure of ICS. (b) Experimental protocol.

METH (purity ≥ 99.1%) was obtained from the National Institute for the Control of Pharmaceutical and Biological Products (Beijing, China). ICS (Figure 1(a)) was purchased from the ZZBIO Co., Ltd. (Shanghai, China), and ML385 was purchased from the Macklin Biochemical Co., Ltd. (Shanghai, China). The antibodies used in this study were TH (AB152, Millipore, Germany), DAT (bs-1714R, Bioss, China), *α*-syn (A7215, Abclonal, China), GFAP (16825-1-AP, Proteintech, China), Iba1 (ab220815, Abcam, USA), Keap1 (60027-1-Ig, Proteintech, China), Nrf2 (66504-1-Ig, Proteintech, China), HO-1 (70081S, CST, USA), NQO1 (ab80588, Abcam, USA), *β*-actin (bs-0061R, Bioss, China), and corresponding secondary antibodies (BL003A and BL001A, Biosharp, China).

### 2.2. Animal Treatments

Male C57BL/6J mice (20-22 g, 8 weeks old) were purchased from the Laboratory Animal Center of Southern Medical University (Guangzhou, China) and housed in a standard animal room with temperature (22 ± 2°C), humidity (55 ± 5%), light cycles (12 h light and 12 h dark), and free access to food and water. All experiments were approved by the Animal Care and Use Committee of Southern Medical University (No. L2017208) and were performed according to ethical standards described in the NIH guidelines. The chronic METH-abuse mice model was established according to the 14-day administration schedule (Table 1), which was determined based on our previous studies [8, 27, 28]. And 10 mg/kg or 30 mg/kg of ICS was administered orally to investigate the intervention effect of ICS on METH-induced neurotoxicity by referring to an earlier study [29]. As shown in Figure 1(b), the animal experiments were divided into two phases.

In the first phase, the mice were randomly divided into five groups (16 mice per group):

1. Control: saline (orally, once a day for four weeks)+saline (saline in place of METH, intraperitoneal injection, according to the 14-day dosing schedule)
2. ICS_H_: 30 mg/kg of ICS (orally, once a day for four weeks)+saline (saline in place of METH, intraperitoneal injection, according to the 14-day dosing schedule)
3. METH: saline (orally, once a day for four weeks)+METH (intraperitoneal injection, according to the 14-day dosing schedule)
4. METH+ICS_L_: 10 mg/kg of ICS (orally, once a day for four weeks)+METH (intraperitoneal injection, according to the 14-day dosing schedule)
5. METH+ICS_H_: 30 mg/kg of ICS (orally, once a day for four weeks)+METH (intraperitoneal injection, according to the 14-day dosing schedule)

In the second phase, the mice were randomly divided into six groups (16 mice per group):

1. Control: saline (intraperitoneal injection, once a week for four weeks)+saline (orally, once a day for four weeks)+saline (intraperitoneal injection, according to the 14-day dosing schedule, but replace METH with saline)
2. METH: saline (intraperitoneal injection, once a week for four weeks)+saline (orally, once a day for four weeks)+METH (intraperitoneal injection, according to the 14-day dosing schedule)
3. METH+ICS_H_: saline (intraperitoneal injection, once a week for four weeks)+30 mg/kg of ICS (orally, once a day for four weeks)+METH (intraperitoneal injection, according to the 14-day dosing schedule)
4. ML385: 30 mg/kg of ML385 (intraperitoneal injection, once a week for four weeks)+saline (orally, once a day for four weeks)+saline (intraperitoneal injection, according to the 14-day dosing schedule, but replace METH with saline)
5. METH+ML385: 30 mg/kg of ML385 (intraperitoneal injection, once a week for four weeks)+saline (orally, once a day for four weeks)+METH (intraperitoneal injection, according to the 14-day dosing schedule)
6. METH+ICS_H_+ML385: 30 mg/kg of ML385 (intraperitoneal injection, once a week for four weeks)+30 mg/kg of ICS (orally, once a day for four weeks)+METH (intraperitoneal injection, according to the 14-day dosing schedule)

All mice were euthanized 24 h after behavioral tests and the brains were harvested for further analysis.

### 2.3. Behavioral Tests

For pole test, the pole (length: 75 cm and diameter: 1.5 cm) was placed on the ground verticality. The mice were placed near the top (5 cm from the top) of the pole facing upwards. The total time taken to reach the bottom of the pole was recorded. For rotarod test, the mice were placed on a wheel and the time of latency to fall was recorded. The speed of the rotarod started from 4 to 40 rpm, and the acceleration rate was 20 rpm/min. Each test was conducted 3 times after 2 days of training. For gait test, the apparatus is a U-shaped runway (length: 60 cm, width: 10 cm, and height: 10 cm). The paper was placed on the bottom of the runway. The mice were allowed to run from one side to the other side. Before the test, the mice were trained for 2 days. In the test trials, the mice forepaws were painted red and hindpaws black using nontoxic dyes. The mice were placed on the runway, and the footprints were acquired. Stride length was measured between each of the forepaw and hindpaw footprints.

### 2.4. Immunohistochemical (IHC) Staining

The mice brains were fixed in 4% paraformaldehyde and then dehydrated in gradient alcohol. After being embedded in paraffin, the brain tissues were sectioned at the coronal plane using a microtome (RM 2235, Leica, Germany). Sections containing substantia nigra (SNc) and caudate and putamen (CPu) areas were subjected to IHC staining. Briefly, the sections were immersed into sodium citrate solution for antigen recovery. The sections were then incubated with the anti-TH antibody for 12 h at 4°C. Thereafter, the targeted proteins were visualized by 3'3-diaminobenzidine Kits (CW2069, CW Bio, China). The images were captured by a microscope (CX23, Olympus, Japan).

### 2.5. Nissl Staining

The Nissl staining was performed by using a Nissl staining kit (G 1434, Solarbio Life Sciences, China). The paraffin-embedded brains were sectioned using a microtome (RM 2235, Leica, Germany). The sections were dewaxed, rehydrated, and then immersed into methylene blue staining solution for 10 min. After being immersed in the Nissl differentiation solution for 3 s, the sections were rinsed in water and then dehydrated in pure alcohol. A microscope (CX23, Olympus, Japan) was used to obtain images.

### 2.6. Western Blot

The striatum tissues were homogenized in a protein extraction buffer (Beyotime, China) containing protease and phosphatase inhibitors. After centrifugation (12,000 g, 10 min, 4°C), the protein supernatant was collected and measured with a Protein Quantitative Analysis kit (Biocolors, China). The protein loading buffer was added to the supernatant and boiled at 99°C for 10 min. The samples were separated by SDS-PAGE and transferred to 0.45 *μ*m PVDF membranes (Millipore, USA). After blocking in 5% nonfat milk at room temperature for 1 h, the membranes were then incubated overnight at 4°C with the following primary antibodies: TH, DAT, *α*-syn, GFAP, Iba1, Keap1, Nrf2, HO-1, NQO1, and *β*-actin. The dilution ratios for all antibodies were 1 : 1000. In the next day, the membranes were incubated with the secondary antibody at room temperature for 1 h, and electrochemiluminescence reagents (Bio-Rad, USA) were added to visualize the immunoblot signals. ImageJ software was used to measure band densities.

### 2.7. Measurement of Dopamine (DA) Levels

The striatum was homogenized in an ice-cold buffer containing 0.01 mM of HClO_4_ and 0.01% EDTA. After centrifugation (20,000 g, 20 min, 4°C), DA levels in the supernatant were assessed by using HPLC.

### 2.8. Dendritic Spine Analysis

Brain tissues were fixed in 4% paraformaldehyde for 6 h. The sections (thick in 200 *μ*m) were acquired using a vibratome (VT1200S, Leica, Germany). Lucifer yellow dye (L453, MA, USA) was loaded in a pipette for injection into the neurons of CPu. The dye was injected into a neuron for 25 min with a 1-3 nA current. The pipettes were removed when the dendritic branches were visualized. Dendritic images were acquired under a confocal microscope (LSM 880, Zeiss Carl, Germany). The numbers of dendritic spines were analyzed using ImageJ software.

### 2.9. Measurement of ROS, MDA, SOD, and GSH Levels

The ROS levels in striatum were measured by using an ELISA assay kit (MEIMIAN, China). Briefly, the striatum was homogenized in ice-cold saline, and the supernatant was collected after centrifugation (3500 rpm, 15 min, 4°C). After a series of incubation and washing, a microplate reader (BioTek, USA) was used to detect the absorbance at 450 nm, which can be used to calculate the ROS levels. The MDA, SOD, and GSH levels in striatum were measured by using commercial kits (Nanjing Jiancheng Bioengineering Institute, China) according to the manufacturer's instructions. The samples from striatum were measured with a microplate reader (BioTek, USA) at 532, 450, and 405 nm for further calculating MDA, SOD, and GSH levels, respectively.

### 2.10. Data Analysis

All experiments were repeated at least four times and data were represented as the mean ± SD. One-way ANOVA (followed by the Tukey HSD and LSD tests) was performed by using SPSS 21 (IBM SPSS, Chicago, United States), and *p* < 0.05 was considered statistically significant.

## 3. Results

### 3.1. ICS Attenuated Neuronal Loss and Improved Behavioral Performance in Chronic METH-Abuse Mice Model

**Figure 2:**
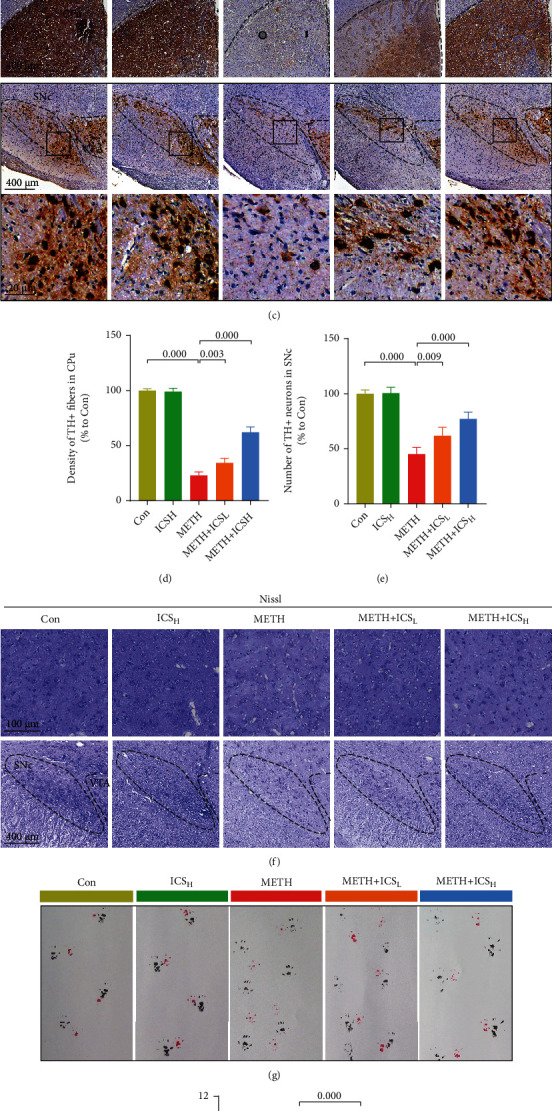
ICS attenuated neuronal loss and improved behavioral performance in chronic METH abuse mice model. (a) The fall latency was determined by rotarod test. (b) The descend time was determined by pole test. (c) Representative micrographs of TH staining in CPu and SNc (scale bar = 400 *μ*m for the low-magnification images and 20 *μ*m for the high-magnification images). (d) Quantification of TH-positive fibers in CPu. (e) Quantification of TH-positive neurons in SNc. (f) Representative micrographs of Nissl staining in CPu (scale bar = 100 *μ*m) and SNc (scale bar = 400 *μ*m). (g) Representative footprint patterns from gait test. (h) Analysis of stride length. *n* = 6 per group for behavioral tests and *n* = 4 per group for IHC staining and Nissl staining.

Rotarod test, pole test, and gait test revealed that chronic METH abuse induced the impairment of motor coordination and balance, while both low (10 mg/kg) and high (30 mg/kg) dosages of ICS could improve these impaired behavioral performances elicited by METH (Figures 2(a), 2(b), 2(g), and 2(h)). We next performed IHC staining of TH to detect the dopaminergic neuron number in SNc and CPu of each group of mice (Figure 2(c)). Quantitative analysis of TH staining showed that both 10 mg/kg and 30 mg/kg of ICS could attenuate the loss of dopaminergic neurons in METH-treated mice (Figures 2(d) and 2(e)). Similarly, the numbers of Nissl-positive cells were increased in SNc and CPU of ICS+METH mice compared to METH mice (Figure 2(f)).

### 3.2. ICS Increased TH, DAT, and DA Levels and Decreased GFAP, Iba1, and *α*-Syn Levels in Striatum of METH Mice Model

**Figure 3:**
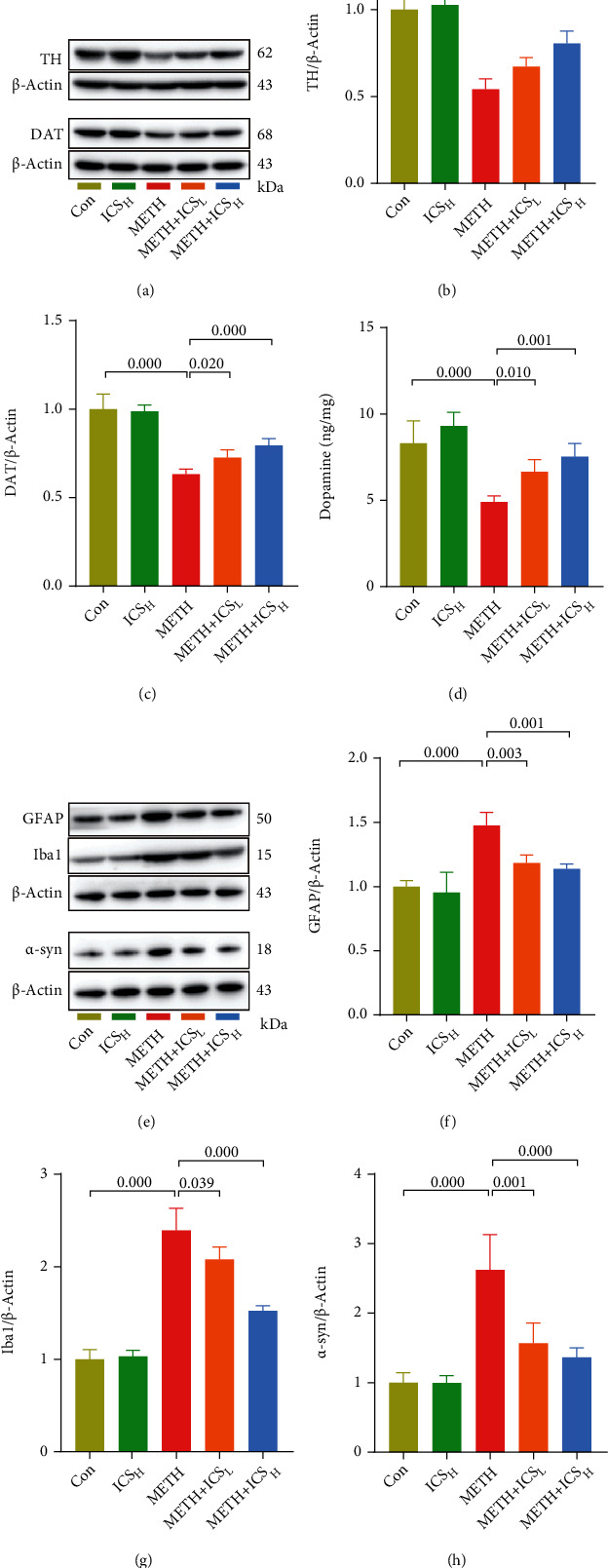
ICS increased TH, DAT, and DA levels and decreased GFAP, Iba1, and *α*-syn levels in striatum of METH mice model. Figures 3(a)–3(c) Representative WB images and quantification of TH and DAT. (d) Measurement of DA levels by using HPLC. Figures 3(e)–3(h) Representative WB images and quantification of GFAP, Iba1, and *α*-syn. *n* = 4 per group.

To further verify the protective effects of ICS on dopaminergic neurons decreased by METH, WB and HPLC analyses were used to detect DAT, TH, and DA levels in striatum of each group of mice, respectively. We found that the levels of DAT, TH, and DA remarkably decreased in METH mice compared to control mice. However, treatment with ICS increased TH, DAT, and DA levels reduced by METH (Figures 3(a)–3(d)). Considering that glial activation and high expression of *α*-syn are manifestations of METH neurotoxicity, we continued to detect the expression of GFAP, Iba1, and *α*-syn in striatum of each group of mice. The WB results suggested that METH-induced high-level expressions of GFAP, Iba1, and *α*-syn were suppressed in ICS-received mice (Figures 3(e)–3(h)).

### 3.3. ICS Alleviated the Abnormalities of Dendritic Spines of Neurons in CPu of METH Mice Model

**Figure 4:**
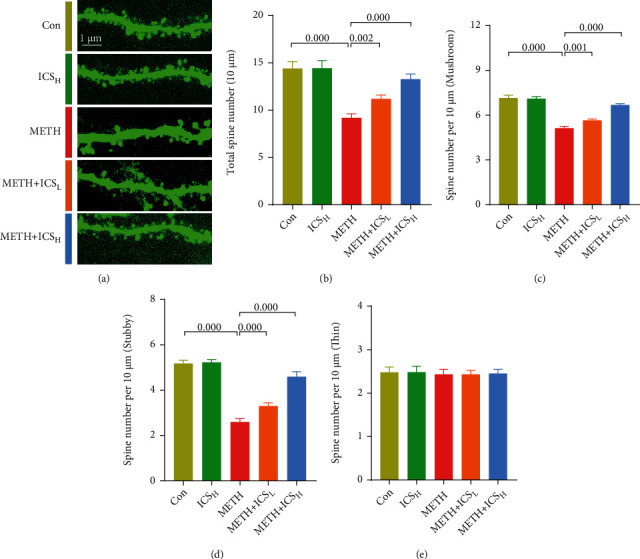
ICS alleviated the abnormalities of dendritic spines of neurons in CPu of METH mice model.  Figure 4(a) Representative images of dendritic spines (scale bar = 1 *μ*m). Figures 4(b)–4(e) Analysis of the numbers of total dendritic spines, mushroom-type dendritic spines, stubby-type dendritic spines, and thin-type dendritic spines. *n* = 4 per group.

We next conducted morphology analysis for dendritic spines of neurons in CPu area. Compared with the control mice, METH-treated mice exhibited decreased numbers of total dendritic spines, mushroom-type dendritic spines, and stubby-type dendritic spines in neurons. Both low and high dosage ICS intervention alleviated the abnormalities of dendritic spines of neurons in CPu of METH mice model (Figures 4(a)–4(d)). In addition, we found that there was no difference in the numbers of thin-type dendritic spines of neurons between each group of mice (Figure 4(e)).

### 3.4. ICS Activated the Keap1-Nrf2 Pathway and Reduced Oxidative Stress in Striatum of METH Mice Model

**Figure 5:**
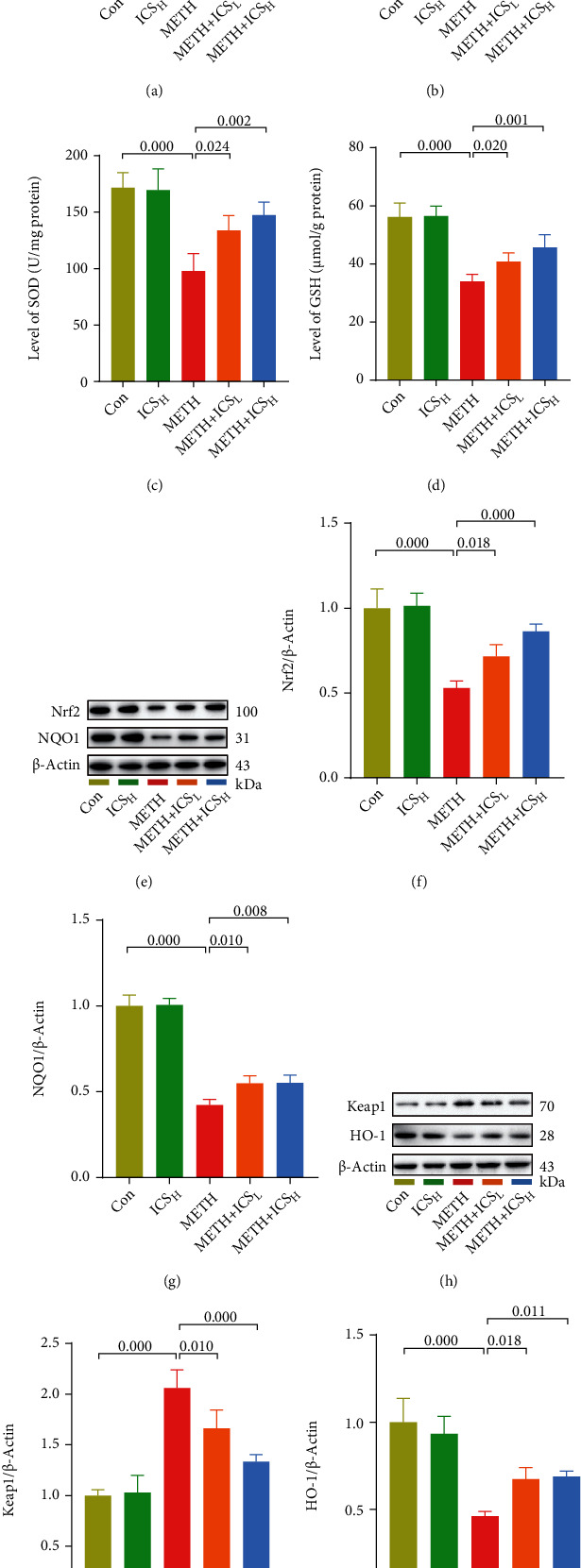
ICS activated the Keap1-Nrf2 pathway and reduced oxidative stress in striatum of METH mice model. Figure 5(a) The ROS levels were measured by using an Elisa kit. Figures 5(b)–5(d) The MDA, SOD, and GSH levels were measured by using commercial kits. Figures 5(e)–5(j) Representative WB images and quantification of Nrf2, NQO1, Keap1, and HO-1. *n* = 4 per group.

Given the crucial role of oxidative stress in METH neurotoxicity, we then investigated whether the Keap1-Nrf2 pathway and its mediated antioxidative stress were countable for the neuroprotective ability of ICS. Here, we found that METH inhibited the Keap1-Nrf2 pathway and elicited a serve oxidative stress response, which was featured by the upregulation of ROS (Figure 5(a)), MDA (Figure 5(b)), and Keap1 (Figures 5(h) and 5(i)) levels and by the downregulation of SOD (Figure 5(c)), GSH (Figure 5(d)), Nrf2 (Figures 5(e) and 5(f)), NQO1 (Figures 5(e) and 5(g)), and HO-1 (Figures 5(h) and 5(j)) levels in striatum of METH mice, whereas the decrease or increase of these indicators was all recovered to some extent in both ICS_L_+METH mice and ICS_H_+METH mice compared to METH mice (Figures 5(a)–5(j)). These data implied that ICS might protect against METH-induced neurotoxicity by activating the Keap1-Nrf2 pathway and reducing oxidative stress.

### 3.5. In the Presence of ML385, an Inhibitor of Nrf2, ICS Failed to Activate the Nrf2-Related Protein Expression and Reduce Oxidative Stress in Striatum of METH Mice Model

**Figure 6:**
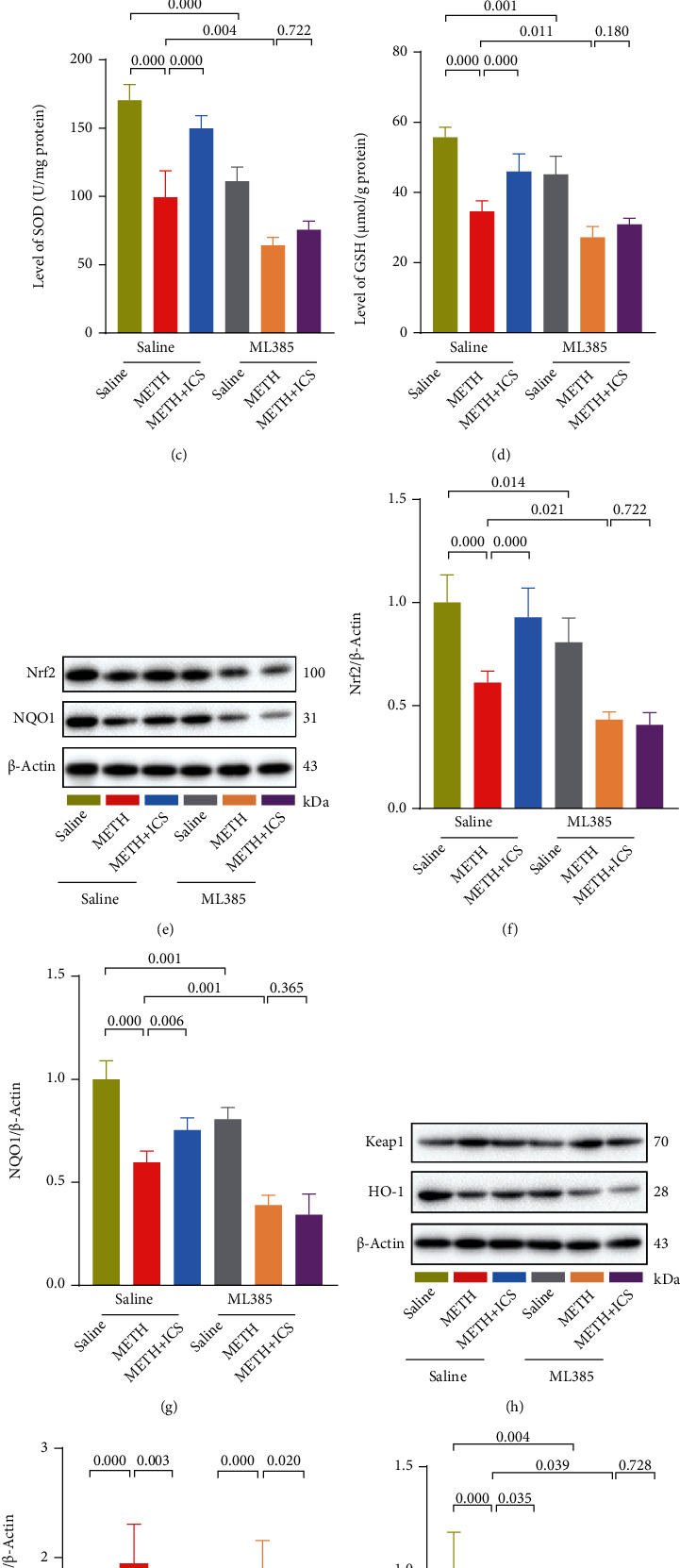
In the presence of ML385, an inhibitor of Nrf2, ICS failed to activate the Nrf2-related protein expression and to reduce oxidative stress in striatum of METH mice model. Figure 1(a) The ROS levels were measured by using an Elisa kit. Figures 6(b)–6(d) The MDA, SOD, and GSH levels were measured by using commercial kits. Figures 6(e)–6(j) Representative WB images and quantification of Nrf2, NQO1, Keap1, and HO-1. *n* = 4 per group.

ML385, an inhibitor of Nrf2 [30], was used to further confirm whether the Keap1-Nrf2 pathway and its mediated antioxidative stress were involved in the protective effect of ICS on METH neurotoxicity. First, we focused on the effect of ML385, ICS, and METH on the Keap1-Nrf2 pathway and oxidative stress response. Consistent with the first phase of the experiment, we found that ICS activated the Keap1-Nrf2 pathway and reduced oxidative stress in striatum of METH mice (Figures 6(a)–6(j)). Besides, the mice treated with ML385 alone exhibited lower levels of Nrf2, NQO1, HO-1, GSH, and SOD but higher levels of ROS and MDA than the control mice. The changes of these indicators were more obvious in ML385+METH mice compared to METH alone mice (Figures 6(a)–6(h) and 6(j)). In addition, we found that the regulation of Keap1 by ICS was not affected in the presence of ML385 (Figures 6(h) and 6(i)). Nevertheless, after inhibiting Nrf2 by ML385, ICS failed to activate the Nrf2-related protein expression and to reduce oxidative stress in striatum of METH mice model (Figures 6(a)–6(h) and 6(j)).

### 3.6. When Nrf2 Was Suppressed, ICS Could Not Attenuate Dopaminergic Neurotoxicity, nor Improve Impaired Behavioral Performance in Chronic METH Abuse Mice Model

**Figure 7:**
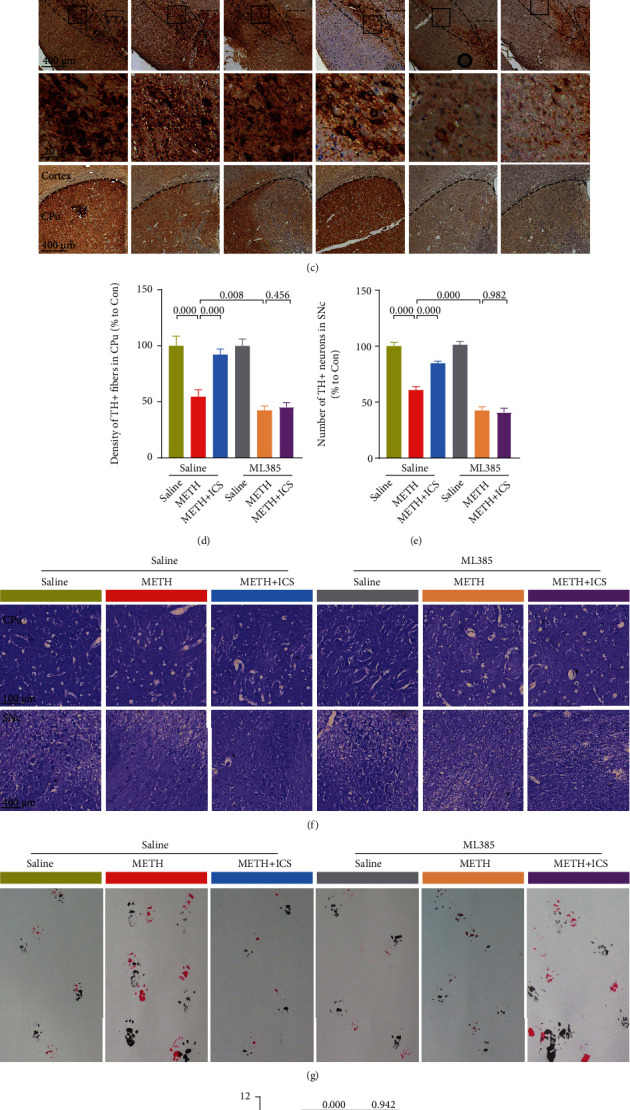
ML385 blocked the protective effects of ICS on neuronal loss and behavioral impairment in METH mice model. Figure 7(a) The fall latency was determined by rotarod test. Figure 7(b) The descend time was determined by pole test.  Figure 7(c) Representative micrographs of TH staining in CPu and SNc (scale bar = 400 *μ*m for the low-magnification images and 20 *μ*m for the high-magnification images).  Figure 7(d) Quantification of TH-positive fibers in CPu.  Figure 7(e) Quantification of TH-positive neurons in SNc.  Figure 7(f) Representative micrographs of Nissl staining in CPu (scale bar = 100 *μ*m) and SNc (scale bar = 400 *μ*m).  Figure 7(g) Representative footprint patterns from gait test.  Figure 7(h) Analysis of stride length. *n* = 6 per group for behavioral tests and *n* = 4 per group for IHC staining and Nissl staining.

**Figure 8:**
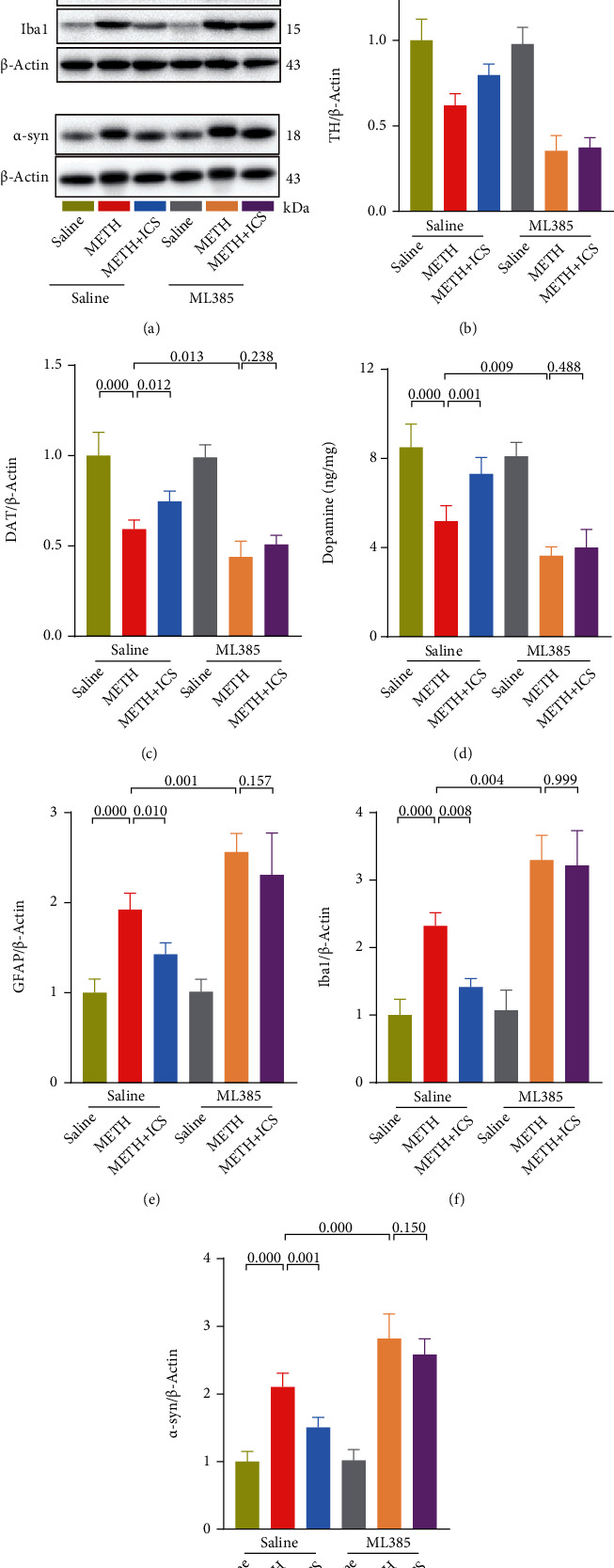
Inhibition of Nrf2 eliminated the regulatory effect of ICS on the abnormal expression of TH, DAT, DA, GFAP, Iba1, and *α*-syn in striatum of METH mice model. (a–c and e–g) Representative WB images and quantification of TH, DAT, GFAP, Iba1, and *α*-syn. (d) Measurement of DA levels by using HPLC. *n* = 4 per group.

**Figure 9:**
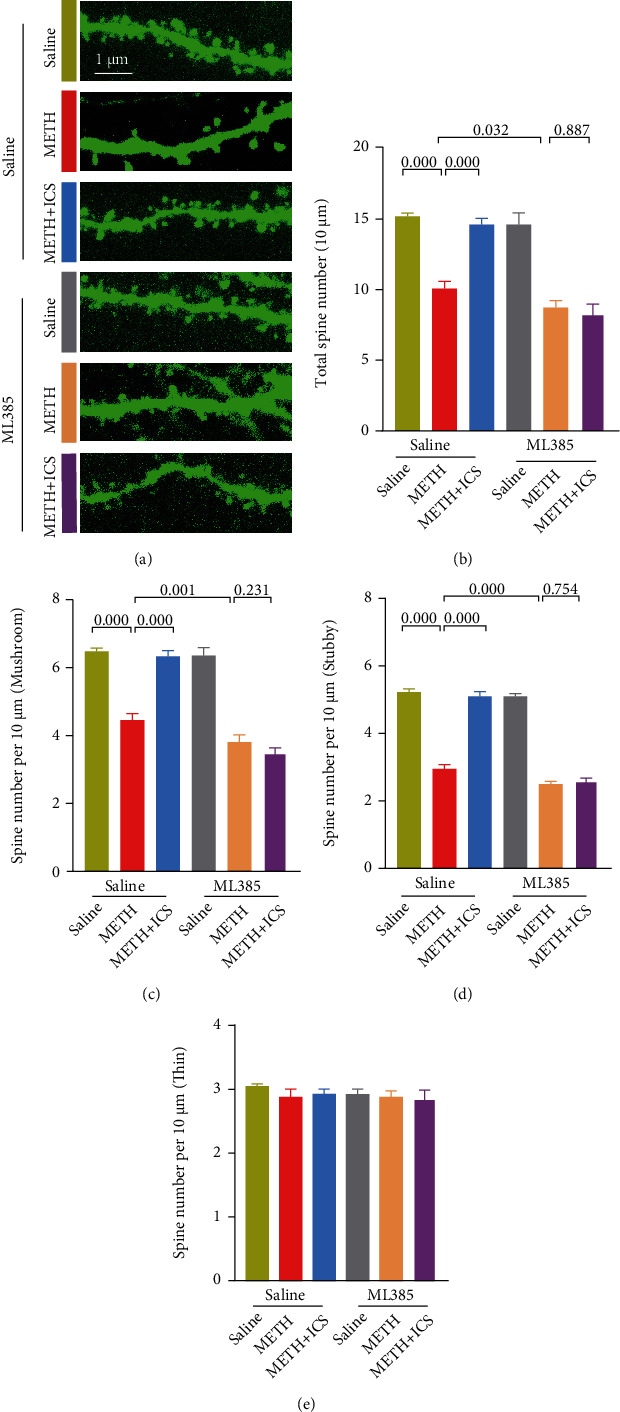
When Nrf2 was suppressed, ICS could not alleviate the abnormalities of dendritic spines of neurons in CPu of METH mice model.  Figure 9(a) Representative images of dendritic spines (scale bar = 1 *μ*m). Figures 9(b)–9(e) Analysis of the numbers of total dendritic spines, mushroom-type dendritic spines, stubby-type dendritic spines, and thin-type dendritic spines. *n* = 4 per group.

Next, we will determine if ICS, when Nrf2 was suppressed, could still attenuate dopaminergic neurotoxicity and improve impaired behavioral performance in chronic METH abuse mice model. Compared with the METH alone group, the indicators that reflected the damage to dopaminergic neurons and motor coordination and balance became more serious in METH+ML385 group. In behavioral performance, the fall latency in rotarod test was lower (Figure 7(a)), the descend time in pole test was longer (Figure 7(b)), and the stride length in gait test was shorter (Figures 7(h) and 7(g)). In morphological and molecular studies, the numbers of TH-positive neurons and Nissl-positive neurons were lower in SNc and CPu (Figures 7(c)–7(f)). The TH, DAT, and DA levels were lower (Figures 8(a)–8(d)), and the GFAP, Iba1, and *α*-syn levels were higher (Figures 8(a) and 8(e)–8(g)). The decreased numbers of total dendritic spines, mushroom-type dendritic spines, and stubby-type dendritic spines in neurons were also more obvious (Figures 9(a)–9(d)). More importantly, while ICS treatment could alleviate the above-mentioned neurotoxicity and behavioral damage induced by METH, however, when Nrf2 was suppressed, ICS failed to show its neuroprotective effects in chronic METH abuse mice model (Figures 7–9). This suggests that the neuroprotective effect of ICS on METH-induced neurotoxicity was achieved by activating the Keap1-Nrf2 pathway.

## 4. Discussion

This study showed that METH could trigger neurotoxicity, which was characterized by dopaminergic neurons loss, glial cell activation, *α*-syn upregulation, DA depletion, and dendritic spines abnormalities. The neurotoxicity is also accompanied by behavioral impairments (specifically, dysfunction of motor coordination and balance). ICS treatment alleviated the above-mentioned neurotoxicity and behavioral impairments induced by METH. We also found that the neuroprotective effect of ICS on METH-induced neurotoxicity was achieved by activating the Keap1-Nrf2 pathway and decreasing the oxidative stress response.

The toxic effect of METH on dopaminergic neurons is one of its most representative neurotoxic manifestations, which has been extensively studied. Due to the high lipid solubility, METH can cross the blood-brain barrier (recent studies suggested that METH can destroy it [31]) and enter the brain parenchyma. METH infiltrates the brain further into dopaminergic neurons through the DAT and exerts its toxic effects there. METH initially enhances DA release and inhibits DA reuptake, thereby activating dopaminergic signals in the reward pathway to trigger reward behavior and subsequently drug addiction. However, with the prolonged METH exposure, neurotoxic effects on dopaminergic neurons occur, specifically manifested as the downregulation of TH and DAT levels and DA depletion [10, 32]. Using positron emission tomography imaging, Volkow et al. [33] found that the DAT density in striatum of METH abusers was decreased compared to those of healthy individuals. Previous studies have also found that METH treatment could reduce the expression levels of TH, DAT, and DA in laboratory animals [6, 8, 34, 35]. Consistent with these studies, we duplicated the dopaminergic neurotoxic phenotype in mice after chronic METH exposure. Considering the various pharmacological properties of ICS, we selected it as a candidate to study whether it could reduce the neurotoxicity induced by METH. We have collected evidence showing that ICS could attenuate the loss of dopaminergic neurons and the decrease of DA concentrations in the METH mice model. The neuroprotective effects of ICS have been mentioned in other animal disease models. Xu et al. [26] demonstrated that ICS could mitigate OGD/R-induced primary hippocampal neuron injury. ICS also remarkably ameliorated beta-amyloid (A*β*) generation and neuronal degradation in APP/PS1 double transgenic mice [29]. Interestingly, a recent study found that human amniotic mesenchymal stem cells could differentiate into dopaminergic neuron-like cells under the influence of ICS [36]. Thus, both this study and previous studies suggest the neuroprotective potential of ICS.

METH-induced dopaminergic neuron damage is similar to the pathological changes of PD, which is an increased risk for METH abusers [37]. In this study, a high-level expression of *α*-syn was observed in striatum of METH mice model, which was consistent with our previous studies [35, 38, 39]. The abnormalities in the dendritic spines of striatal neurons were observed in PD [40]; therefore, the pathological changes of dendritic spines of neurons in CPu were analyzed after METH exposure. We found that METH caused abnormal dendritic spine morphology and spine number. We then detected the motor coordination and balance ability of METH-treated mice through pole test, rotarod test, and gait test. Compared with the control mice, mice treated with METH exhibited poorer behavioral performance. The above results further proved that METH could induce PD-like symptoms [35]. Surprisingly, unlike the protective effects of ICS on the Alzheimer's disease (AD) model [41–44], we found in this study that ICS could alleviate METH-induced high-level expression of *α*-syn, dendritic spines abnormalities, and dysfunction of motor coordination and balance.

Oxidative stress plays an essential role in METH-induced neurotoxicity [45], and it always has crosstalk with inflammatory response, apoptosis, autophagy, and other molecular pathological mechanisms [4, 12, 46]. ROS is a by-product of aerobic metabolism, including superoxide anion, hydroxyl radical, and hydrogen peroxide. Under the dynamic equilibrium between the oxidation system and antioxidant system, excess ROS will be cleared in time. However, when the body's antioxidant system is weakened or ROS is greatly increased, oxidative stress damage will occur. Due to the strong oxidative effect, METH abuse will result in oxidative stress damage. Zeng et al. [46] pointed out that METH augmented intracellular ROS levels and downregulated the level of glutathione peroxidase 1 (GPX1) and SOD1. This severe oxidative stress further induced autophagy and apoptosis in human SH-SY5Y neuroblastoma cells and rat striatum.

Nrf2 exerts an antioxidant effect by dissociating from Keap1 and transferring into nuclear, where it interacts with the antioxidant response element and activates the expression of antioxidant enzymes. When Nrf2 nuclear translocation and its downstream gene expression be inhibited, it will cause redox imbalance and oxidative stress injury [47]. Conversely, activating the Keap1-Nrf2 pathway by increasing the expression and effect of Nrf2 will play a protective role against disease states [48, 49]. Unfortunately, multiple exposures to METH increased Keap1 expression but decreased Nrf2 expression and thus downregulated downstream antioxidant enzyme expressions like HO-1 and glutamyl-cysteine synthetase-*γ* [18]. Besides, METH not only enhanced ROS production but also inhibited the Keap1-Nrf2 pathway, thus resulting in damage to neurons in ventral tegmental area of rats [19]. In line with these previous results, in this study, we also found that chronic METH abuse inhibited the Keap1-Nrf2 pathway and elicited oxidative stress. Moreover, ICS exhibited positive effects on activating the Keap1-Nrf2 pathway and attenuating oxidative stress in striatum of METH mice model, implying the involvement of the Keap1-Nrf2 pathway and oxidative stress in ICS-regulated METH neurotoxicity.

We next used ML385, an inhibitor of Nrf2, to further confirm whether the Keap1-Nrf2 pathway and oxidative stress were involved in the protective effect of ICS on METH neurotoxicity. Just as expected, in the presence of ML385, ICS failed to activate the Keap1-Nrf2 pathway and to reduce oxidative stress in striatum of METH mice. More importantly, ML385 blocked the protective effects of ICS on neuronal loss, dendritic spines abnormalities, and behavioral impairments induced by METH. Also, inhibition of Nrf2 eliminated the regulatory ability of ICS on the abnormal expression of TH, DAT, DA, GFAP, Iba1, and *α*-syn. These results proved that ICS could attenuate the METH-induced neurotoxicity via modulating the Keap1-Nrf2 pathway and the oxidative stress. Although previous studies have shown that ICS might achieve antioxidant effects by activating Nrf2-related protein expression and decreasing ROS levels in several diseases [50–52], we collected evidence for the first time that ICS could combat METH-induced neurotoxicity and PD-like symptoms through mediating the classic antioxidant Keap1-Nrf2 pathway and oxidative stress.

For decades, accumulative evidence from preclinical studies has shown that many natural plants and their active ingredients have preventive and therapeutic effects on neurodegenerative diseases. For example, in the 1-methyl-4-phenyl-1,2,3,6-tetrahydropyridine- (MPTP-) induced PD mice model, chlorogenic acid, ursolic acid, *Tinospora cordifolia*, and *Mucuna pruriens* could exhibit antiapoptotic, anti-inflammatory, antioxidant, or other pharmacological properties, which contribute to protection against neurotoxicity elicited by MPTP [53–57]. Similarly, our study found that ICS, a type of flavonoid and one of the main active ingredients of the traditional Chinese medicine *Epimedium*, could confer neuroprotection against METH-induced neurotoxicity via the Keap1-Nrf2 pathway activation and subsequent antioxidant and anti-inflammatory reinforcement.

While the mechanism of how Nrf2 dissociates from Keap1 is unclear, studies have shown that several kinases, such as advanced protein kinase B (AKT) and extracellular signal-regulated kinase (ERK), may be involved in Nrf2 activation and nuclear translocation. Lv et al. [58] found that Licochalcone A could enhance Nrf2 nuclear translocation and HO-1 expression through AKT and ERK activation in *tert*-butyl hydroperoxide-treated RAW 264.7 cells. The protective effect of sesamin on ulcerative colitis was also involved in the activation of AKT/ERK and subsequent enhancement of Nrf2 signaling [59]. The limitation of this study was that we did not investigate the prime target molecules directly affected by ICS in the METH exposure model. Therefore, the exact mechanism by which ICS activates the Keap1-Nrf2 pathway remains to be answered in the future.

Besides, the anti-inflammatory role of the Keap1-Nrf2 pathway has been fully verified and widely recognized. The nuclear factor kappa B (NF-*κ*B) is one of the most concerned transcription factors in inflammatory pathways. It has been suggested that there is complex crosstalk between the Nrf2 and NF-*κ*B pathways. A review written by Bellezza et al. [60] pointed out that compounds that suppress NF-*κ*B signaling could activate the Nrf2 pathway, and activated NF-*κ*B could also stimulate the Nrf2 pathway, which could inhibit NF-*κ*B activity conversely. Previous studies indicated that ICS could attenuate lipopolysaccharide-induced neuroinflammation by regulating the NF-*κ*B pathway [21, 22]. Consistently, in this study, ICS activated the Keap1-Nrf2 pathway suppressed by METH to decrease glial cell activation. However, this positive influence was blocked when Nrf2 was inhibited.

In sum, the major findings of this work are that ICS can attenuate the METH-induced neurotoxicity and PD-like behavioral impairments via activating the Keap1-Nrf2 pathway. Although further research is needed to dig deeper into the actual molecular targets of ICS, it is undeniable that the current results imply the potential value of ICS to reduce the neurotoxicity of METH abusers.

## Figures and Tables

**Table 1:** The METH (mg/kg) administration schedule.

Day	1	2	3	4	5	6	7	8	9	10	11	12	13	14
8 : 00	1.0	1.0	1.0	1.0	1.5	1.5	2.0	2.0	2.5	3.0	3.5	4.0	4.5	5.0
10 : 00				1.0	1.5	1.5	2.0	2.0	2.5	3.0	3.5	4.0	4.5	5.0
12 : 00				1.0	1.5	1.5	2.0	2.0	2.5	3.0	3.5	4.0	4.5	5.0
14 : 00		1.0	1.0	1.0	1.5	1.5	2.0	2.0	2.5	3.0	3.5	4.0	4.5	5.0

## Data Availability

The original contributions presented in the study are included in the article.
